# Days, rather than hours of care, mediate the effect of older adults’ cognitive performance on the burden of informal caregivers

**DOI:** 10.1002/alz.092861

**Published:** 2025-01-09

**Authors:** Lucas Nogueira de Carvalho Pelegrini, Leticia Fernanda Palma, Laís Rebecchi, Karen Letícia Pulgatti, Pedro Henrique Moreira Victoriano, Danilo Barroso de Sousa, Marina Mantellatto Grigoli, Cecilia Patricia Popolin, Vanessa Alexandre da Silva, Sabrina Dorta de Oliveira, Patricia Regina Manzine, Marcia Regina Cominetti

**Affiliations:** ^1^ Federal University of São Carlos, São Carlos, SP Brazil; ^2^ Federal University of Sao Carlos, São Carlos Brazil; ^3^ Federal University of Sao Carlos, Sao Carlos Brazil; ^4^ Federal University of São Carlos, São Carlos Brazil; ^5^ Federal University of Sao Carlos, Sao Carlos, SP Brazil; ^6^ Global Brain Health Institute, Dublin, Dublin Ireland

## Abstract

**Background:**

Informal care of older adults impacts the lives of millions worldwide. Critically, low‐ and middle‐income countries have the highest proportion of dementia costs related to informal care. Research suggests that older adults’ cognitive decline is associated with burden among caregivers, which corroborates the worsening in caregivers’ quality of life and mental health. Understanding factors related to this relationship may contribute to creating strategies to reduce the caregiver’s burden. This study analyzes whether hours and days dedicated to care mediate the relationship between older adults’ cognitive performance and caregiver burden.

**Method:**

Participants were informal caregivers (n = 92) of healthy older adults (n = 74) and persons living with Alzheimer’s Disease (PLwAD, n = 18) from a middle‐income country (Brazil). The assessment comprised the following instruments: a sociodemographic questionnaire, Zarit burden scale, Memory Complaint Scale‐caregiver’s version (MCS), Pfeffer Functional Activities Questionnaire (FAQ), and IQCODE. ACE‐R was used to assess older adults’ cognitive performance.

**Result:**

Caregivers were mostly female (72.8%), daughters (42.4%), or wives (43.5%), who were dedicated to caring, on average, 7.6 (±9.1) hours per day and 5 (±2) days per week. PLwAD caregivers had higher scores on the Zarit Scale (t = ‐5.35; p<0.001), MCS (t = ‐11.54; p<0.001), FAQ (t = ‐14.31; p<0.001), IQCODE (t = ‐6.36; p<0.00). They also provided more hours of care (t = ‐3.97; p<0.001; 95%CI [‐15.89 to ‐4.93]), for more days per week (t = ‐2.30; p = 0.028; 95%CI [‐2.65 to ‐0.16]). Mediation analysis indicated that cognitive performance had a total effect of ‐0.374 (p<0.001; 95%CI [‐0.52 to ‐0.23]) on caregivers’ burden. When controlling for days of care, the direct effect of cognitive performance on the burden was ‐0.3251 (p<0.001; 95%CI [‐47 to ‐0.18]), and there was an indirect effect of ‐0.0698 (95%BCaCI [‐0.14 to ‐0.01]) through the mediation of days of care. Considering hours of care per day, the indirect effect of cognitive performance on the burden was not significant (95%BCaCI [‐0.21 to 0.005]), which suggests it does not mediate this relationship.

**Conclusion:**

Our findings suggest that days dedicated to care mediate the effect of older adults’ cognitive performance on caregivers' burden. These findings underscore the importance of considering the temporal aspects of caregiving in developing strategies to alleviate caregiver burden.

